# Histologic Study of Abdominal Skin Treated With Mechanical Dermal Micro‐Coring Technology for Minimally Invasive Skin Removal

**DOI:** 10.1111/jocd.70323

**Published:** 2025-07-02

**Authors:** Ashish C. Bhatia, Karl Napekoski, Jill Edgecombe, David Weir, Rachelle Winkle, Rod Rohrich

**Affiliations:** ^1^ Associate Professor of Clinical Dermatology Northwestern University, Feinberg School of Medicine Chicago Illinois USA; ^2^ Adjunct Associate Professor of Clinical Dermatology Rush University School of Medicine Chicago Illinois USA; ^3^ Co‐Director of Dermatologic, Laser & Cosmetic Surgery and Medical Director for Dermatologic Research, Oak Dermatology Naperville Illinois USA; ^4^ Dermatopathologist at Midwest Diagnostic Pathology Illinois USA; ^5^ Independent Consultant Atlanta Georgia USA; ^6^ Mr. Weir Is a NP in Private Practice at Dallas Plastic Surgery Institute Dallas Texas USA; ^7^ Ms. Winkle Is an RN in Private Practice at Dallas Plastic Surgery Institute in Dallas Texas USA; ^8^ Dr. Rohrich Is a Plastic Surgeon in Private Practice at Dallas Plastic Surgery Institute Dallas Texas USA; ^9^ A Clinical Professor of Plastic Surgery‐Baylor College of Medicine Houston Texas USA; ^10^ A Professor of Plastic Surgery University of Texas Southwestern Medical Center Dallas Texas USA

**Keywords:** histology, mechanical dermal micro‐coring technology (MCT), minimally invasive skin removal, skin removalabdominoplasty

## Abstract

**Background:**

Mechanical Dermal Micro‐Coring Technology (MCT; Ellacor System) achieves skin tightening and wrinkle reduction through direct mechanical excision of skin cores using hollow needles and collagen stimulation via the wound‐healing response.

**Aims:**

To evaluate histopathology and immunohistochemistry of the skin and subcutaneous tissue after a single and multiple treatments with MCT.

**Patients/Methods:**

In this single‐center pilot study, 6 female patients scheduled to undergo abdominoplasty were divided into 2 cohorts. Subjects in cohort 1 (safety cohort) received 1 MCT treatment at a depth of 4 mm, 5 mm, and 7 mm, each administered at 1 of 3 unique test areas, with tissue sampling/abdominoplasty 30 days after treatment. Subjects in cohort 2 received 1, 2, or 3 4 mm‐depth treatments at 1 of 3 unique test sites, with 30‐day intervals for > 1 treatment. Tissue sampling/abdominoplasty occurred 90 days after initial treatment. Histopathology was performed at a central laboratory, and biopsies were evaluated using H&E, Herovici, and Movat stains by blinded evaluators.

**Results:**

A robust increase in new collagen compared to the control tissue was observed for 1 to 3 treatments in all samples. There was no evidence of inflammation or scarring, consistent with earlier preclinical and clinical histology.

**Conclusions:**

MCT‐associated histological changes confirm that in addition to skin removal, treatment results in an increase in collagen and homogenization of the dermis in the treated area.

## 1 Introduction

Tradition nonsurgical skin tightening methods have relied on either thermocoagulation with energy or mechanical disruption with microneedling to stimulate the wound‐healing process and promote the formation of collagen. The effect on skin laxity is indirect with these technologies. Excess skin is not removed; rather, the wound‐healing process alone is responsible for the treatment effect and aesthetic outcomes. This indirect approach has limitations, and oftentimes patients experience suboptimal results, in particular if they have excess skin in combination with volume deficits prior to treatment.

In contrast, Mechanical Dermal Micro‐Coring Technology (MCT; Ellacor [Cytrellis Biosystems Inc., Woburn, MA]) [1] utilizes excisional skin remodeling to both remove skin and stimulate the wound‐healing response. Skin removal is minimally invasive. Skin cores are excised by hollow needles and evacuated from the needle by suction. Once treatment is complete, an immediate impact is apparent as skin has been mechanically removed. This initial effect is complemented by the wound‐healing response, which takes place over time. Patients most often receive between 1 and 3 treatments to achieve the desired effect, with the treatment number contingent upon baseline severity.

Due to the novel mechanism of action for MCT, histologic analyses have been carried out as part of multiple clinical studies, including one that established 500 μm as the safety threshold for coring needle diameter. Full‐thickness skin micro‐columns at diameters < 500 μm permit treatment without scarring in patients with FST (I‐III) [2]. Above this threshold, a subset of patients experience some indication of fibrosis on histology and evidence of persistent redness and some visible removal sites. Based on these findings, the MCT device was developed with 400‐μm diameter coring needles. Histologic examination of tissue has not been limited to studies of needle diameter. Overall, the histologic response to MCT has been examined in 29 patients in three separate safety studies and in 59 patients as part of a prospective multicenter clinical trial. No evidence of scarring was observed in these studies with coring needles 400 μm in diameter for up to 90 days in patients with Fitzpatrick skin types I‐IV [2, 3, 4].

**FIGURE 1:**
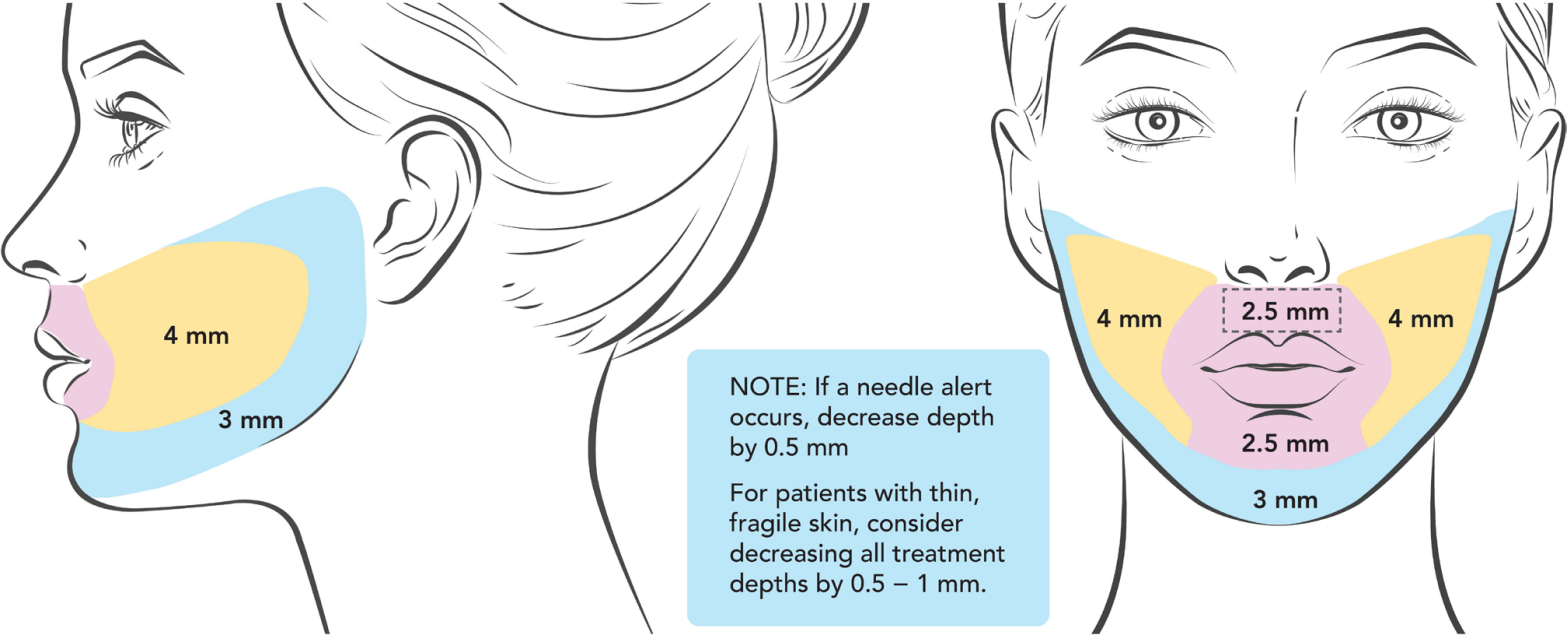
Recommended treatment depths for on‐label use of MCT treatment of the face.

The coring needles, which penetrate the epidermis and dermis, can be used at depths up to 4.0 mm, adjustable in 0.5‐mm increments. In clinical practice, multiple treatments are often administered to achieve optimal effects (Figure 1). In this study, we examined histopathology and immunohistochemistry of the skin and subcutaneous tissue following a single treatment and multiple treatments with micro‐coring technology at multiple depths.

## 2 Methods

In this single‐center prospective study, 6 female patients age 18 years or older who were scheduled to undergo abdominoplasty were enrolled. Patients were not excluded based on Fitzpatrick skin type. Patients with a history of abnormal wound healing or keloid formation, as well as patients taking medications or with medical conditions that would worsen bleeding or bruising were not enrolled. Patients were divided into two cohorts. Patients in Cohort 1 (safety cohort) received a single MCT treatment at each of three unique test areas in the abdomen. At each of the three treatment areas, treatment was administered at 1 of 3 depths: 4 mm, 5 mm, and 7 mm for a total of three treatments for each patient in Cohort 1 (Table 1). A non‐treated control area was also designated. At 30 days following this single treatment, abdominoplasty was performed. Treated subjects in Cohort 1 were followed for safety assessments at 7, 14, 21, and 30 days post treatment and were monitored for adverse events (AEs).

**TABLE 1:** Treatment cohorts and assessments.

	Cohort 1	Cohort 2
Treatment	Single treatment Area 1: 4 mm Area 2: 5 mm Area 3: 7 mm	3 treatments, 30 days apart^a^ Area 1: 3 treatments Area 2: 2 treatments Area 3: 1 treatment
Assessments	Safety assessments at 7, 14, 21, and 30 days post‐treatment Tissue sampling for histology taken 30 days post‐treatment	Histology evaluations at 30 days after Area 3 treatment (Day 90)

^**a**^ All treatments were 4 mm depth.

Patients in Cohort 2 also had 3 unique test areas in the abdomen. At each unique test area, treatment was administered 1, 2, or 3 times at single treatment depth (4 mm), for a total of three treatment areas per patient (Table 1). In instances when multiple treatments were administered, administration occurred at 30‐day intervals: treatments were administered on Day 0, Day 30, and Day 60. A non‐treated control area was also designated. At 90 days following the initial treatment (30 days following the third treatment), abdominoplasty was performed. Cohorts are summarized in Table 1. Patients in Cohort 2 did not return to the office for follow‐up between MCT treatment and abdominolasty; however, patients were encouraged to report any AEs to the treating investigator and were questioned about AEs at the time of abdominoplasty. For both cohorts, an intra‐procedural bleeding assessment was conducted based on a 4‐point severity scale (none, mild, moderate, severe) for each of the treatment areas.

Histologic evaluations were carried out for all treatment areas to evaluate tissue after treatment and ensure there was no evidence of scarring. Histopathology of the excised abdominal tissue was performed at a central pathology laboratory, and biopsies were evaluated using H&E, Herovici, and Movat stains by blinded evaluators. Increases in collagen noted in the results are based on qualitative observations and are not directly measured. This study was IRB‐approved by Allendale IRB, and patients were treated in accordance with the principles outlined in the Declaration of Helsinki.

## 3 Results

A total of 6 female patients, median age 37.8 years (range 3–58) were enrolled and completed the study. A total of 4 patients with FST II skin were enrolled, 2 in each cohort; 1 patient with FST III skin was enrolled in Cohort 2; and 1 patient with FST VI was enrolled in Cohort 1. For Cohort 2, all treatment visits for Day 30 (time of second treatment) and Day 60 (time of third treatment) were within 19 to 31 days and 41 to 52 days, respectively. Day 90 visits, when abdominoplasty was carried out, took place between 77 and 89 days post initial treatment. For Cohort 1, all patients underwent abdominoplasty 28 days following initial treatment.

**FIGURE 2:**
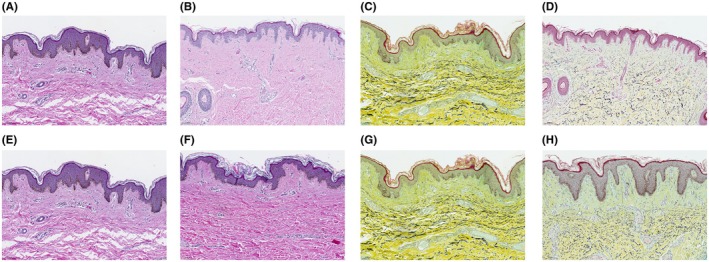
Assessment of multiple treatment depths. H&E (A, B, E, F) and Movat stained (C, D, G, H) specimens from 2 patients in Cohort 1 who received treatments at multiple depths. Samples shown are from the control area (A, C, E, G) and 30 days following treatment at a depth of 7 mm (B, D, F, H). Magnification 30×.

H&E stain demonstrated a healthy, intact epidermis across all 3 treatment areas in both cohorts, with the epidermis in the control area and treated areas appearing intact and healthy. An increase in collagen was observed at all treatment depths compared to control in all subjects without evidence of scar formation (Figure 2). Moreover, Movat staining demonstrated preserved elastic fiber distribution and no histologically visible scar formation at all treatment depths examined (Figure 2C,D,G,H).

**FIGURE 3:**
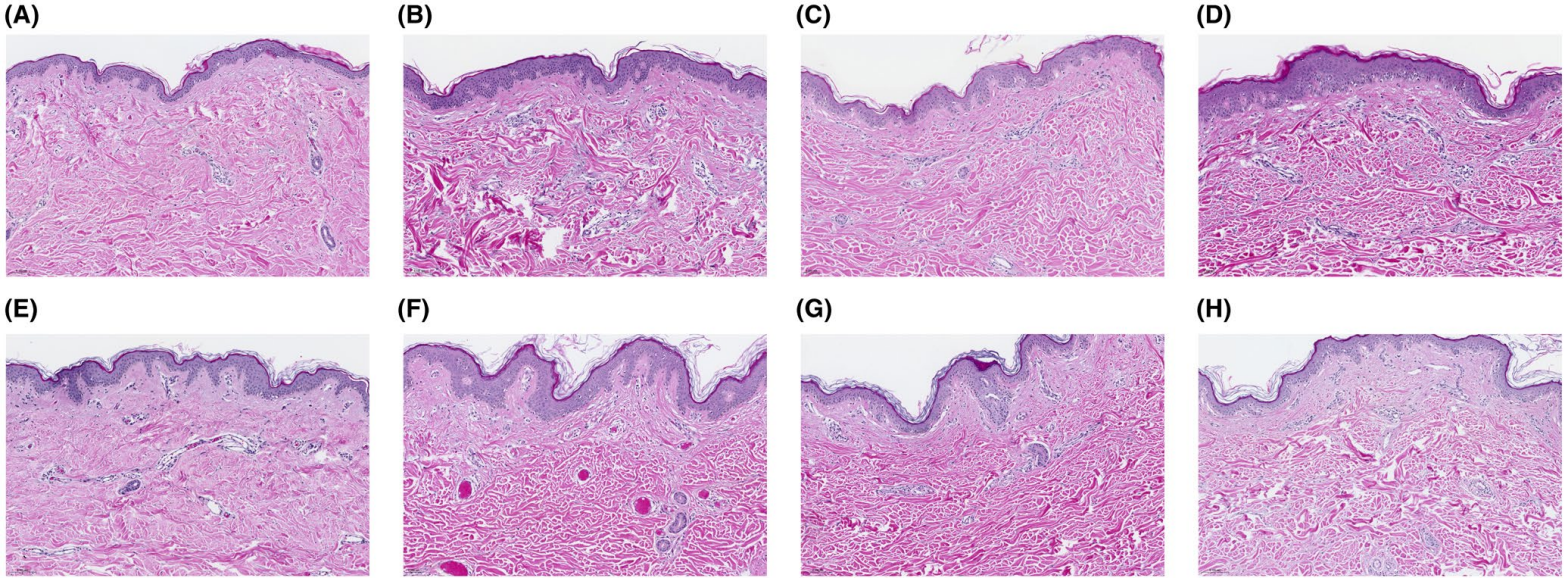
Assessment of multiple treatment sessions. H&E stain from 2 subjects in Cohort 2. Samples are from the control area (A, E) at 90 days following a single treatment (B, F), 60 days following the second of 2 treatments (C, G), and 30 days following 3 treatments (D, H). An increase in collagen was observed at all treatment time points. Magnification 30×.

**FIGURE 4:**
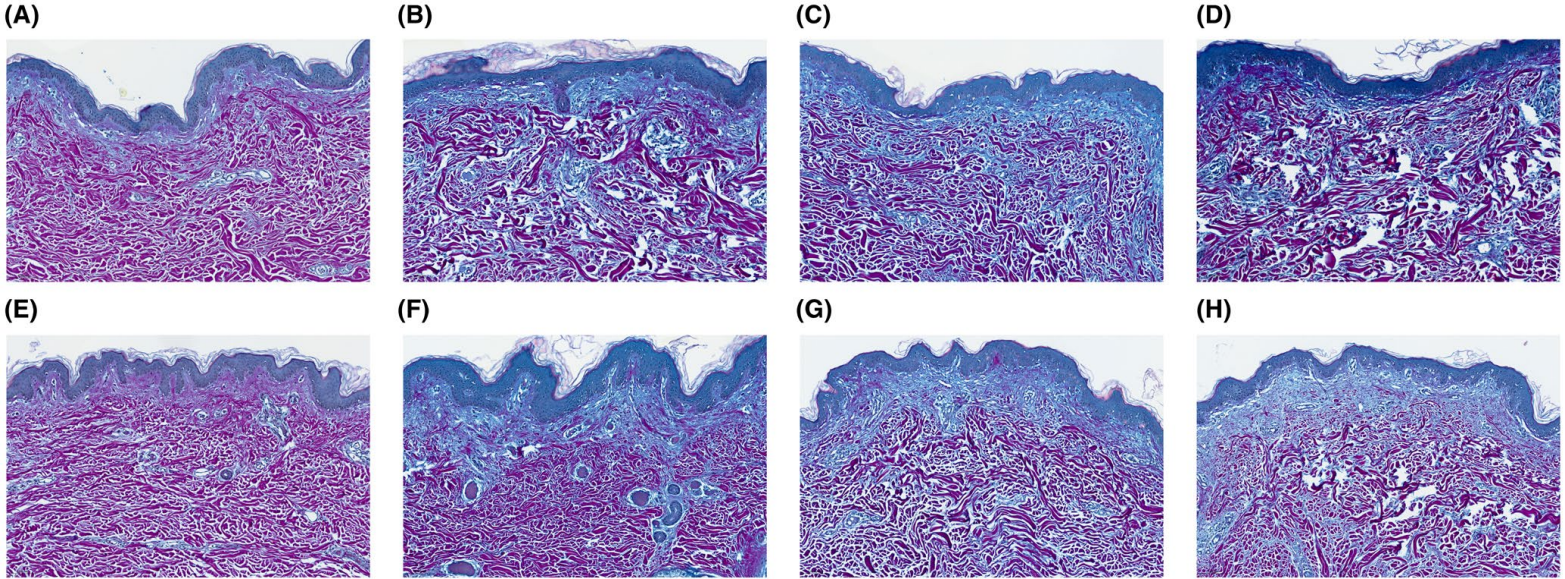
Increase in collagen in the reticular dermis and papillary dermis. Herovici stain from 2 subjects in Cohort 2 who received serial treatments. Samples are from the control area (A, E), 90 days following a single treatment (B, F), 60 days following the second of 2 treatments (C, G), and 30 days following 3 treatments (D, H). Magnification 30×.

Compared to the control areas, the reticular dermis in the treated areas demonstrated changes consistent with reorganization and homogenization of collagen fibers. A robust increase in new collagen deposition was observed for 1 to 3 treatments compared to the untreated control in all subjects (treatments administered ~30 days apart at 4‐mm depth; Figure 3). Herovici stain highlighted magenta‐staining mature collagen fibers within the reticular dermis and revealed an appreciable increase in new collagen compared to the control tissue in the papillary dermis (Figure 4). In Cohort 1, an approximately 25% to 50% increase in new (immature) collagen fibers was observed within the dermis compared to the control tissue at all treatment depths. An approximately 50% to 100% increase in new (immature) collagen fibers within the dermis was observed in Cohort 2 compared to control for all non‐control treatments.

Across cohorts, all treatment‐related AEs were typical and expected reactions. Adverse events were restricted to redness and flaking and were mild in severity (Table 2). Mild bleeding was reported for all subjects in both cohorts following treatments. Across both cohorts, all bleeding reported was mild. In Cohort 1, the 3 subjects reported mild bleeding for all treatment depths, and mild bleeding was associated with each treatment in all 3 patients in Cohort 2. No evidence of trauma or significant inflammation was observed in the histologic analysis.

**TABLE 2:** Adverse Events recorded during the study for Cohort 1 and Cohort 2.

Treatment parameter	Reported AE	Cohort 1 count (*N* = 3)
4‐mm depth	Flakiness/flaking skin	2
	Redness	3
5‐mm depth	Flakiness/flaking skin	2
	Redness	3
7‐mm depth	Flakiness/flaking skin	2
	Redness	3
**Treatment parameter**	Reported AE*	**Cohort 2 count (*N* = 3)**
1 treatment	Flakiness/flaking skin	0
	Redness	3
2 treatments	Flakiness/flaking skin	0
	Redness	6
3 treatments	Flakiness/flaking skin	0
	Redness	9

* No AEs were reported for the control areas.

## 4 Discussion

These findings indicate that MCT results in a substantial increase in collagen and elastin at treatment depths up to 7 mm and with 1 to 3 treatments at a treatment depth of 4 mm with no indication of scarring. This observation of increased collagen without evidence of inflammation is consistent with both safety and clinical efficacy studies, which consistently demonstrate safety at depths up to 4 mm in facial areas [3, 4]. In earlier histology studies, the primary objective was to determine the optimal inner diameter for the coring needles; thus, this is the first study to more systematically address different treatment depths. Clinically, MCT can be applied at multiple treatment depths depending on the area being treated (Figure 1). The finding that minimal evidence of scarring is apparent at depths up to 7 mm may be relevant for future indications outside of the face such as scar treatment, management of fibrotic tissue, or treatment of lax skin on the body, should a deeper depth ever be considered for these purposes.

While this is a preliminary study with a small number of patients, the findings indicate that repeat treatment of the same area is not associated with an increased risk of inflammation or scarring. While this study's population in Cohort 2 is too small to draw any conclusions around the additive benefit of each treatment in terms of collagen induction, a qualitative, incremental increase can be appreciated in Figures 3 and 4 for each treatment. This is consistent with molecular data showing increased expression of COlA1, COL3A1, and elastin at 45 days following a single treatment (at the time of a second treatment) and upregulated further 45 days following a second treatment (90 days following the first treatment) [5]. Clinically, in the authors' experience, most patients require at least two treatments to achieve an optimal effect and sufficient skin removal, and three treatments are generally needed for patients with significant laxity at baseline. These histology data show that on the tissue level, there is no indication that three treatments leads to abnormal tissue effects such as scarring or inflammation. This is important information for clinicians, as it suggests that this number of treatments could be safely applied without increased risk.

Finally, the robust collagen induction observed across all treatment groups is supportive of a dual mechanism for MCT in which skin removal is complemented by a wound‐healing response whereby neocollagenesis and neoelastogenesis lead to aesthetic improvement over time following treatment. Further, homogenization of collagen and elastin is seen throughout the dermis as a result of these treatments.

The limitations of this study include the small patient population and the lack of quantitative comparisons between groups. In future studies, quantitative methods in a larger study population could be used to determine the impact of a single versus serial treatments on collagen expression in the skin. Furthermore, this analysis was done on abdominal skin, rather than facial skin, which could partially limit the applicability of results.

## 5 Conclusion

The histological changes that occur following MCT treatment in patients undergoing abdominoplasty surgery confirm that in addition to skin removal, treatment results in an increase in collagen in the treated area. Substantial neocollagenesis was observed at treatment depths up to 7 mm and for 1 to 3 treatments, with no evidence of inflammation or scarring.

## Author Contributions

D.W., R.W., and R.R. performed the procedures. K.N. is a pathologist and independently processed tissue samples. A.C.B. and K.N. reviewed histology images. J.E. and A.C.B. designed the research study. R.R. assisted in research development and drafting of the manuscript. All authors reviewed and approved the manuscript.

## Conflicts of Interest

Dr. Bhatia is a Consultant and Medical Director for Cytrellis Biosystems Inc.; Jill Edgecombe is a former employee of Cytrellis Biosystems Inc.; Mr. Weir is a consultant for Revance and Cytrellis Biosystems Inc.; Dr. Rohrich has received research support from and served as a consultant for Allergan/AbbVie, the Musculoskeletal Transplant Foundation (MTF), and Galderma; served as a consultant, investigator, and speaker for In Mode; served as a consultant for Evolus; has received research support from Merz, Cytrellis, Rion, and Teoxane; and receives book royalties from Thieme Publishers and instrument royalties from Eriem Surgical (Micrins).

## Data Availability

Research data are not shared.

## References

[1] U.S. Food and Drug Administration , Cytrellis Dermal Micro‐Coring System July 12, 2023, https://www.accessdata.fda.gov/cdrh_docs/pdf20/K202517.pdf.

[2] A. H. Champlain , C. M. DiGiorgio , D. Zurakowski , F. H. Sakamoto , and R. R. Anderson , “Wound Healing After Fractional Skin Harvesting,” Dermatologic Surgery 48, no. 10 (2022): 1083–1088, 10.1097/DSS.0000000000003552. 36036977

[3] L. Gfrerer , S. L. Kilmer , J. S. Waibel , R. G. Geronemus , and B. S. Biesman , “Dermal Micro‐Coring for the Treatment of Moderate to Severe Facial Wrinkles,” Plastic and Reconstructive Surgery. Global Open 10, no. 10 (2022): e4547, 10.1097/GOX.0000000000004547. 36262685 PMC9575956

[4] J. N. Pozner , S. L. Kilmer , R. G. Geronemus , M. Jack , J. A. Burns , and M. S. Kaminer , “Cytrellis: A Novel Microcoring Technology for Scarless Skin Removal: Summary of Three Prospective Clinical Trials,” Plastic and Reconstructive Surgery. Global Open 9, no. 10 (2021): e3905, 10.1097/GOX.0000000000003905. 34729291 PMC8556055

[5] J. S. Waibel , “Effectiveness of Dermal Micro‐Coring in Achieving Minimally‐Invasive Skin Tightening (MIST): Gene Expression Analysis Results,” in Presented at: ASDS Annual Meeting (American Society for Dermatologic Surgery, November 2‐5, 2023).

